# Linear accelerator (linac) downtime analysis assisted with a Large Language Model (LLM)

**DOI:** 10.1002/acm2.70400

**Published:** 2025-11-27

**Authors:** Yeochan Youn, Joseph B. Schulz, Ethan Stolen, Karl Farrey, Emil Muresan, Siyong Kim, James J. Sohn

**Affiliations:** ^1^ Data Science Institute University of Chicago Chicago Illinois USA; ^2^ Department of Radiation and Cellular Oncology University of Chicago Chicago Illinois USA; ^3^ Department of Medical Physics University of Wisconsin Madison Wisconsin USA; ^4^ Department of Radiation Oncology Virginia Commonwealth University Richmond Virginia USA

**Keywords:** downtime, linear accelerator, LLM, machine performance, time series decomposition

## Abstract

**Background:**

Linear accelerators (LINACs) are critical components of modern radiation therapy, requiring consistent operational performance to ensure uninterrupted patient care. Unplanned downtime not only disrupts clinical workflows but can significantly impact treatment efficacy. Traditional approaches to LINAC reliability analysis have often focused on specific components rather than comprehensive performance patterns. The advent of artificial intelligence and large language models (LLMs) offers new opportunities for analyzing complex, unstructured maintenance data to extract meaningful insights that can inform maintenance strategies and improve clinical operations.

**Purpose:**

This study aims to analyze long‐term operational performance of LINACs by investigating 10 years of maintenance records from three VarianTrueBeam LINACs. We sought to identify fault patterns, quantify downtime durations, determine the most vulnerable components, and evaluate the impact of the COVID‐19 pandemic on maintenance practices.

**Methods:**

We analyzed 1584 maintenance reports from three Varian TrueBeam LINACs spanning 4–13 years of operational use. Our analysis workflow consisted of three main stages: (1) Data normalization involved extracting and cleaning reports from multiple versions of service documents into a uniform tabular structure; (2) Report classification utilized an LLM with custom prompt engineering to categorize each report into predefined work type and failure type categories; and (3) Descriptive analysis examined patterns and trends in machine performance, including time series decomposition to identify seasonal trends and detailed analysis of service events requiring external technician involvement.

**Results:**

All LINACs consistently maintained operational downtime within the 5% threshold agreed upon with the vendor, with only one instance approaching this limit in 2021. Collimation systems, control hardware, and power systems accounted for the highest proportion of maintenance cases. Replacement and repair were the most common work categories. No consistent increasing trend in failure frequency with machine age was observed, and seasonal pattern analysis showed weak seasonality. Field service engineer (FSE) visits have increased steadily over time, with replacement tasks most frequently requiring external technician support. Post‐2020 maintenance showed increased average work hours in most categories compared to pre‐2020, particularly for replacement‐related activities.

**Conclusions:**

Our findings provide insights into the operational performance of LINACs over time, with implications for clinical budget management and maintenance scheduling. The data analysis methodology and LLM‐based classification techniques developed in this study demonstrate an effective approach to analyzing historical maintenance records that could be applied at other institutions.

## 1 INTRODUCTION

Ensuring the consistent performance and availability of linear accelerators (LINACs) is critical for maintaining uninterrupted radiation therapy services. Unplanned downtime of these devices not only disrupts clinical workflows but can also negatively impact treatment efficacy and patient outcomes. Studies have shown that treatment interruptions may lead to decreased local control rates, with even 1 day of interruption potentially decreasing local control by 1.4% in head and neck cancers.^1^ The Association of Physicists in Medicine (AAPM) Task Group (TG) 314 has underscored the importance of fault recovery in radiation therapy, providing detailed guidance on proactive measures, immediate response protocols, and safe resumption of treatment after unplanned downtime.^2^

Modern LINACs represent intricate integration of mechanical, electrical, and software systems, each requiring specialized maintenance to ensure safe and effective operation. While manufacturers typically guarantee 95%–98% uptime in service contracts, achieving this benchmark requires effective management of both preventative maintenance and rapid response to unplanned failures. The American College of Radiology (ACR) and AAPM Technical Standard affirms the central role of qualified medical physicists in LINAC oversight, including assessment of machine performance and implementation of quality assurance programs.^3^ Previous research on LINAC reliability has often focused on specific components rather than comprehensive analysis of overall performance patterns. The AAPM TG‐100 report introduced Failure Mode and Effects Analysis (FMEA) as a systematic approach to identify potential failure modes, evaluate their impacts, and prioritize risk mitigation strategies in radiation therapy, providing valuable guidance for analyzing the various failure modes affecting LINAC operations.^4^

In clinical practice, medical physicists must balance technical aspects of machine management with practical considerations such as budget constraints and patient scheduling. The economic impact of unscheduled downtime is significant, as highlighted by Dunscombe et al. and Pozo et al., necessitating data‐driven approaches to maintenance planning and resource allocation.^5^, ^6^ Furthermore, the COVID‐19 pandemic introduced additional challenges to LINAC maintenance, including supply chain disruptions and modified service protocols. The advent of artificial intelligence and machine learning technologies offers new opportunities for analyzing complex maintenance data, with large language models (LLMs) demonstrating capacity for natural language understanding and classification tasks that can assist in extracting meaningful patterns from unstructured service reports.^7^

For example, one recent study demonstrated how a LLM was used to classify patient self‐reported free‐text inputs across major U.S. health systems and showing that a general‐pupose LLM achieved comparable performance to a specialized classifier.^8^ In a broader industrial review, the diverse applications of LLMs including predictive maintenance, fault classification, and text classification in industrial settings were summarized and highlighting their transformative potential in industrial use‐cases.^9^ Meanwhile, a systematic review of natural language processing (NLP) approaches applied to industrial maintenance records found that unstructured text reports are increasingly being analyzed via NLP techniques to extract failure‐patterns, root‐cause signals and maintenance trends in equipment operations.^10^

To our knowledge, however, such approaches have not yet been applied to the comprehensive maintenance‐log analysis of LINAC systems within a clinical radiotherapy context. This study aims to comprehensively analyze 10 years of maintenance records from three Varian TrueBeam (Varian Medical Systems, a Siemens Healthineers Company, Palo Alto, CA) LINACs to identify patterns in machine failures, quantify downtime durations, determine the most vulnerable components, and evaluate the impact of external factors, such as the COVID‐19 pandemic on maintenance practices. By utilizing a LLM to classify and analyze maintenance reports, we seek to develop a methodological framework that can be applied across institutions to improve LINAC reliability, optimize maintenance scheduling, and ultimately enhance the quality and consistency of radiation therapy services.

## 2 METHODS

**FIGURE 1:**
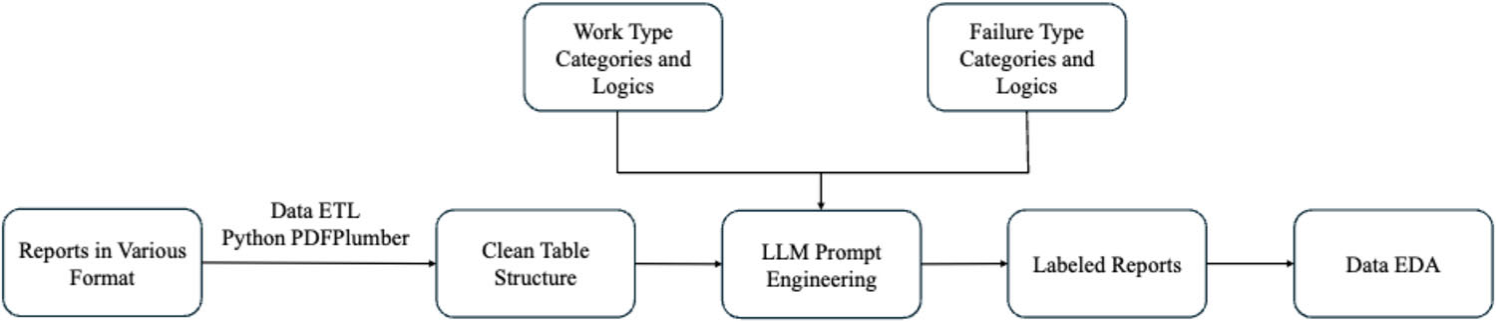
Overview of the data processing and analysis workflow. The pipeline proceeds from left to right, beginning with extraction and structuring of raw service reports using Python‐based ETL tools. Cleaned tabular data is then passed through a large language model (LLM) labeling process, guided by predefined work type and failure type logic. The resulting labeled reports are used for exploratory data analysis (EDA) and visualization.

This study focused exclusively on maintenance and failure records from a Varian TrueBeam LINAC, one of the most widely utilized systems in modern radiation therapy.^11^ At our institution, 1584 maintenance reports were available for three TrueBeam LINACs: LINAC 1 is approximately 12 years of operational use and involved in around 600 reports, LINAC 2 is used around 13 years and recorded in 860 reports, and LINAC 3 is 4 years with 160 reports. The analysis workflow consisted of three main stages. First, data normalization, where reports from the TrueBeam LINACs were extracted from multiple versions of Varian customer service reports and institutional maintenance report using Python and cleaned into a uniform tabular structure. Second, report classification, where the subject and description fields extracted from the data pipeline in the first step were classified into predefined work type and failure type categories using a custom prompt engineering framework powered by a LLM, enabling more efficient data analysis and visualization. Finally, descriptive analysis was performed by integrating the normalized data from the first stage with the LLM‐classified results from the second stage to identify patterns and trends in machine performance. An overview of this process is illustrated in Figure 1, which summarizes the data flow and classification logic used throughout the study.

### 2.1 Data processing

The LINAC maintenance records were archived in multiple versions of Varian customer service report and institutional maintenance report with nonstandardized field structures and naming conventions. A customized Extract, Transform, Load (ETL) pipeline was implemented to execute structured data transformation for enabling comprehensive analysis. PDFplumber (V.0.11.5) and PyMuPDF(V 1.26.5) Python library were used to parse text from each report.^12^ Text extraction logic was adapted to handle various field layouts and to identify key‐value pairs across different templates. The cleaned data were mapped to a predefined tabular schema that ensures consistency across all report types. The final structure includes the following fields: work order ID, machine ID, subject, description, malfunction start time, machine release time, time in, time out, downtime hours, site hours, travel hours, total work hours, report source, and file name (Table 1). This unified schema facilitated downstream classification and analysis tasks, including LLM‐based report category labeling and exploratory data analysis (EDA).

**TABLE 1:** Description of dataset columns. Provides definitions for each column in the processed dataset, outlining the meaning and context of the extracted information used in the analysis.

Field name	Description
Work Order ID	A unique identifier assigned to each maintenance report by the vendor
Machine ID	A unique identifier for each individual LINAC machine
Subject	The report title or summary line provided by the technician or vendor
Description	Detailed notes describing the issue and the corrective actions performed
Malfunction Start Time	Timestamp indicating when the malfunction or issue initially occurred
Machine Release Time	Timestamp indicating when the machine was returned to clinical service
Time in	The time at which the service engineer arrived on site
Time out	The time at which the service engineer completed work and departed
Downtime Hours	Total number of hours the machine was nonoperational due to the issue, out of total clinical operation hours
Site Hours	Duration the engineer was physically present on site performing service
Travel Hours	Time spent by the engineer traveling to the service location
Total Work Hours	Combined total of site hours and travel hours
Report Source	Origin of the report (e.g., internal form or generated form)
File Name	The name of the original digital file from which the report was extracted

### 2.2 Failure types and work types

A consistent taxonomy was required before machine‐learning methods could be applied to the vendor reports, so separate schemas for “failure type” (the subsystem that malfunctioned) and “work type” (the action performed) were developed. Failure types were seeded from the major categories in the Varian TrueBeam Technical Reference Manual, general clinical experience, and then refined. Three iterative review rounds resolved ambiguous cases, and the final schema comprised the 12 mutually exclusive failure types (Table 2). Work type was derived inductively from the vendor reports based on repeated verbiage to encompass the general role of a service engineer, followed similar iterative review, and yielded nine work type classes (Table 3). Multiple work types were allowed to be assigned to a single vendor report.

**TABLE 2:** Failure types from a Varian TrueBeam with description and example.

Failure type name	Description
Beam Generation	Components responsible for creating, accelerating, and bending the initial electron beam, including the electron gun, linear accelerator, bending magnet, and RF power source (Klystron/Magnetron).
Collimation System	Systems that define the final shape of the radiation beam, including primary jaws, MLC, target carousel, and flattening filters/scattering foils.
Gantry Motion/Structure	The mechanical system responsible for rotating the gantry, including drive motors, bearings, position sensors (resolvers/encoders), and the structural frame.
Imaging System (KV/MV)	Hardware used for patient setup and verification imaging, including the kV x‐ray source, kV flat‐panel detector, MV EPID, and associated retractable arms.
Treatment Couch	The patient support system, including its motorized axes (longitudinal, lateral, vertical, rotation, pitch, roll), control pendants, and structural components.
Control Hardware	The distributed network of physical electronic controllers (Supervisor, Nodes), processing boards, and safety interlock circuit hardware managing machine operations.
System Networks	Communication infrastructure connecting system components, including CAN bus, Ethernet networks, HSSB, and associated wiring/connectors.
Operator Console/UI	The operator's interface components, including the main control console buttons/switches, workstation PC, monitors, keyboard/mouse, and the primary software applications (Treatment, Imaging, Service).
Cooling System	Systems managing the thermal environment of critical components, primarily the water‐cooling system (chiller, pumps, flow sensors) and specialized gas systems (e.g., SF_6_ for waveguide).
Power System/Distribution	Components managing the input and distribution of electrical power, including the modulator cabinet, main breakers, high‐voltage circuits, UPS, and power conditioning.
Ancillary Room Systems	Supporting equipment within the treatment room, such as positioning lasers, in‐room cameras (CCTV/Optical Guidance), and room monitors.
Safety Systems	Components specifically designed for personnel and equipment safety, such as emergency stop buttons, door interlocks, collision detection sensors, and radiation monitoring systems.

* BGM: Beam generation and monitoring; CAN: Controller area network; HSSB: High‐speed serial bus; MLC: Multi‐leaf collimator; MV EPID: Megavoltage electronic portal imaging device; RF: Radio frequency; UPS: Uninterruptible power supply.

**TABLE 3:** Work type categories from a Varian TrueBeam with description and example.

Work type name	Description
Replacement	When the primary action involves swapping a faulty, worn, or outdated component.
Calibration	Work focused on tuning, aligning, verifying, or adjusting machine parameters, positions, or outputs.
Maintenance	Routine care, scheduled activities like cleaning, lubrication, inspections, fluid changes (PMP or PM).
Investigation	Diagnosing a fault, reviewing logs, performing tests, inspecting components, identifying root cause.
Repair	Fixing an issue without replacing a major component (e.g., cleaning contacts, reseating connections, adjusting).
Software IT	Actions involving software (updates, rebuilds), system configuration changes, server management (reboot), data management, or network/IT tasks.
Installation	Installing new hardware systems, accessories, or performing mandated upgrades (STBs).
Support	Providing information, remote assistance, consultation, or fulfilling an information request.
Travel	When driving or flying to a site.

* CE: Clinical Engineer; KVS: Kilovoltage source; MPC: Machine performance check; PMP or PM: Preventive maintenance program; STB: Service technical bulletin; STN: Station.

### 2.3 LLM‐based report category labeling

To enable detailed and scalable analysis of the service reports, we employed a LLM‐based labeling approach. LLM classified reports into relevant categories based on the report subtitle and detailed work note.

Since each report can include multiple failures and corresponding tasks performed, the LLM assigned multiple labels to each report for both failure type and work type. In this process, the GPT‐4o‐mini model was utilized due to its computational efficiency with adequate performance in classification tasks.^13^ Additionally, prompt engineering techniques were applied to enhance contextual understanding and improve labeling performance. The prompting techniques employed in the analysis included the following sub‐sections.

#### 2.3.1 Few‐shot prompting

Few‐shot prompting, introduced by Brown et al., involves providing a language model with a several number of input–output examples directly in the prompt, typically by prepending them before the final input query.^13^ This technique allows LLMs to infer the task from context alone, without requiring additional gradient updates.

#### 2.3.2 Chain‐of‐thought prompting

Chain‐of‐thought (CoT) prompting, introduced by Wei et al., augments each example in a prompt with an explicit step‐by‐step reasoning process leading to the final answer.^14^ This strategy enables LLMs to generate intermediate reasoning steps, improving performance on tasks that require multi‐hop or logical reasoning.

#### 2.3.3 Instruction prompting

Instruction prompting, as surveyed by Liu et al., frames a task by providing natural language instructions that describe the desired behavior of the model.^15^ This approach leverages the pretraining knowledge of language models to follow commands when explicitly told what to do. Instruction prompts are often concise and task‐agnostic, making them flexible across a wide range of applications.

#### 2.3.4 Role prompting

Role prompting, introduced by Kong et al., enhances zero‐shot reasoning by instructing the language model to assume a specific identity or role (e.g., a domain expert, teacher, or assistant) before generating a response.^16^ This technique leverages the model's alignment with natural human interactions and its ability to condition outputs on role‐specific behavior.

#### 2.3.5 Output formatting prompting

Output formatting prompting, as described by Hsieh et al. involves guiding language models to produce responses in a specified structure or format, such as JSON objects or tables.^17^ This approach enhances consistency and downstream usability by explicitly specifying the desired output schema within the prompt.

After the LLM generated the labeled dataset, the classification performance was evaluated by comparing the LLM‐assigned labels against manually curated labels, created independently by one medical physicist and one experienced medical physics assistant. A random sample of 48 reports was selected for evaluation. The LLM achieved an accuracy of 0.90 for work type classification and 0.95 for failure type classification, demonstrating strong agreement with expert human annotations. The code example used in this study is available at https://github.com/YeochanY/LINAC‐Downtime‐Analysis/tree/main.

### 2.4 Exploratory data analysis

As noted in Table 1, downtime hours were calculated based on our institution's regular clinic hours which operates from 6 a.m. to 6 p.m. Downtime hours are aggregated yearly for each LINAC machine. A downtime percentage threshold of 5% was set based on agreement between vendor and clinic for reasonable limit that would not critically impact clinical operations. Based on the work type and failure type labeled by a LLM, the number of reports corresponding to each work type and failure type was calculated.

For intuitive pattern analysis, time series decomposition was applied, and data were separated into three components which are trend, seasonal, and residual. The trend component captures the long‐term movement or overall direction of the data, reflecting underlying patterns such as growth or decline. The seasonal component identifies repeating short‐term patterns that occur at regular intervals, such as monthly cycles. In this study, a period of 12 months was selected for the cycles, as the observed data utilized in the time series decomposition consists of monthly observations. Lastly, the residual component represents irregular fluctuations or noise that cannot be explained by the trend or seasonality, capturing random variations or unexpected events within the data. Together, these components provide a clearer understanding of the different factors influencing the time series. Decomposition was performed using an additive model, where the observed data are assumed to be the sum of these three components, as expressed in Equation (1):

(1) $$y_{t}=T_{t}+S_{t}+R_{t}$$

where $y_{t}:\;\mathrm{Observed}\;\mathrm{time}\;\mathrm{series}\;\mathrm{at}\;\mathrm{time}\;T_{t}\;\mathrm{is}\;\mathrm{trend}\;component$, $T_{t}:\;\mathrm{Trend}\;\mathrm{component}$, $S_{t}:\;\mathrm{Seasonal}\mathrm{component}$, and $R_{t}:\;\mathrm{Residual}\;\mathrm{component}$.

Additionally, a separate analysis was conducted for services requiring FSEs by examining the “description” and “travel hours” columns in Table 1. A report is considered as requiring FSEs if recorded travel hours were greater than zero or if travel‐related activities were mentioned in the detailed notes. Finally, a comparison of work hours for the common work types before and after the COVID‐19 pandemic was performed. The calculated work hours included both the technician's travel time and the on‐site service hours spent resolving issues at the clinic. The data were segmented into two periods: before and including the year 2020, and after 2020, reflecting the period when the COVID‐19 pandemic significantly impacted supply chains and clinical operations.

## 3 RESULTS

Comparing the results of the LLM‐based classification against manual labeling, the LLM‐based classification achieved an accumulated accuracy of 0.90 (95% CI: 0.88–0.92) for work type classification and 0.95 (95% CI: 0.94–0.96) for failure type classification.

**FIGURE 2:**
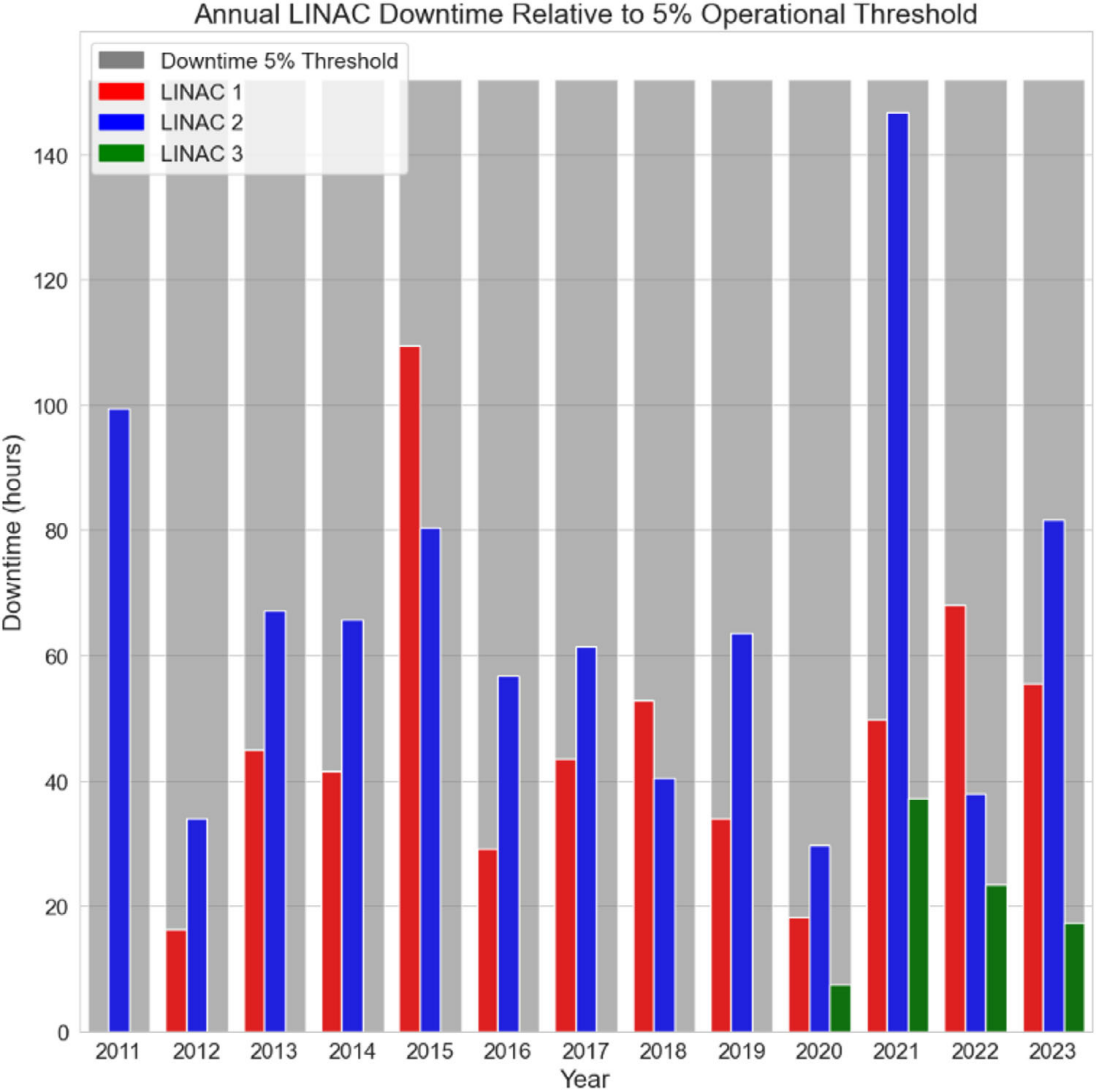
Annual downtime hours for each LINAC compared to the 5% operational threshold. The grey bars represent the 5% threshold for each year. Downtime for LINAC 1, LINAC 2, and LINAC 3 are depicted in red, blue, and green, respectively.

Annual downtime peaked in 2021, with LINAC 2 recording 147 h, just below the 5% downtime threshold of 152 h (Figure 2). Average annual downtime hours were 47 h for LINAC 1 from 2012 to 2023, 67 h for LINAC 2 from 2011 to 2023, and 21 h for LINAC 3 from 2020 to 2023.

**FIGURE 3:**
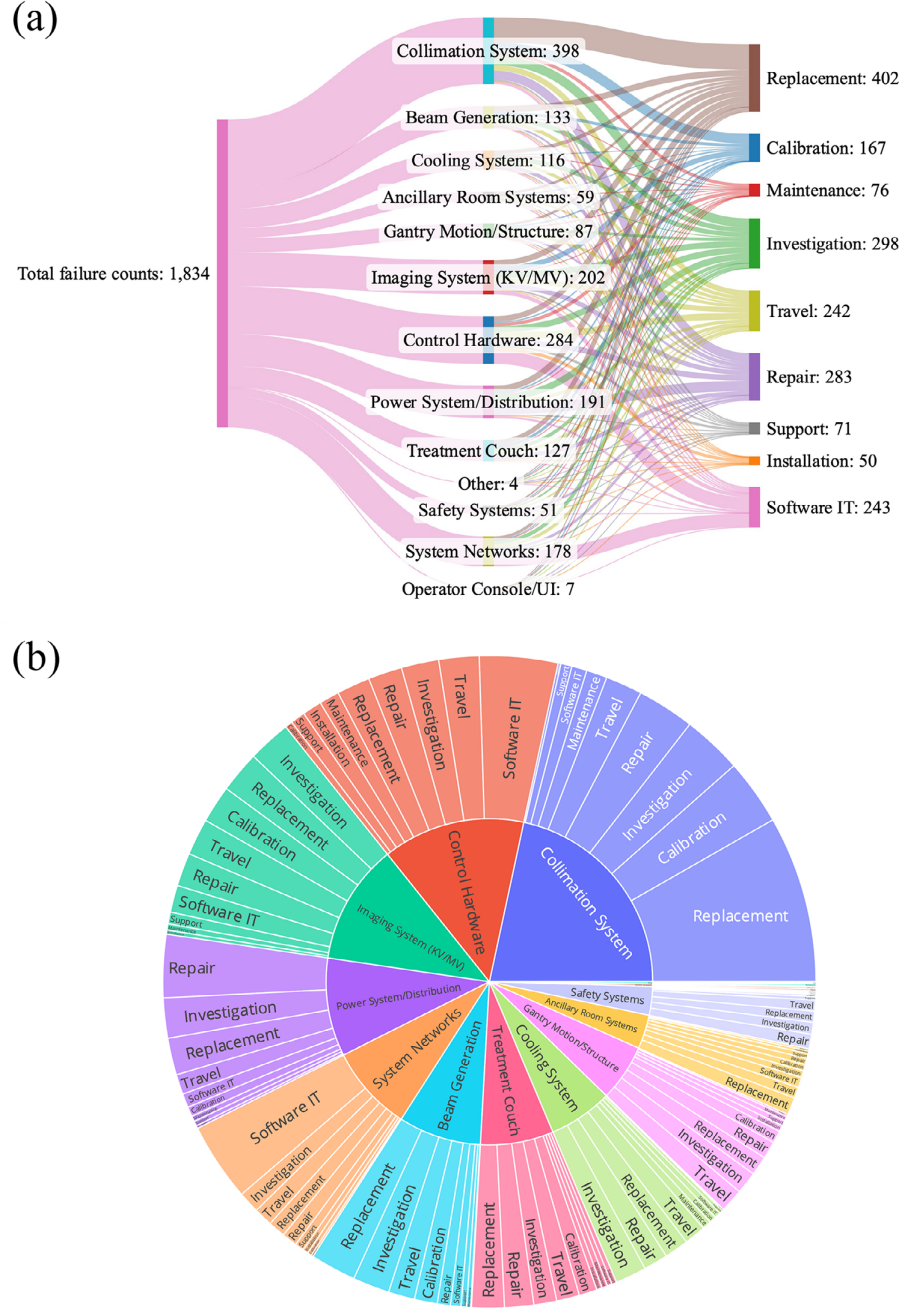
Summary of failure types and corresponding work actions performed on LINAC machines. (a) A total of 1834 failure cases were identified from 1584 service reports. The graph displays the frequency of each failure type, and the number of work actions performed to address them. (b) Chart illustrating the distribution of work types carried out within each failure category.

Categorized report analysis revealed that the collimation system had the highest frequency of failures, comprising 22% of total maintenance cases, followed by control hardware at 15%, power distribution at 10%, and system networks at 10% (Figure 3a). Within the collimation system, the most common maintenance actions were replacement at 38%, calibration at 15%, and investigation at 14% (Figure 3b). Control hardware frequently required software IT interventions at 27%, travel at 14%, and investigation at 13%. The imaging system primarily involved investigation at 18%, replacement at 17%, and calibration at 15%. Repairs dominated the power system with 32%, along with investigation at 20% and replacement at 19%. System networks mostly required replacement at 22%, investigation at 16%, and repair at 15%. Beam generation, cooling, and treatment couch systems often involved replacements and investigations.

**FIGURE 4:**
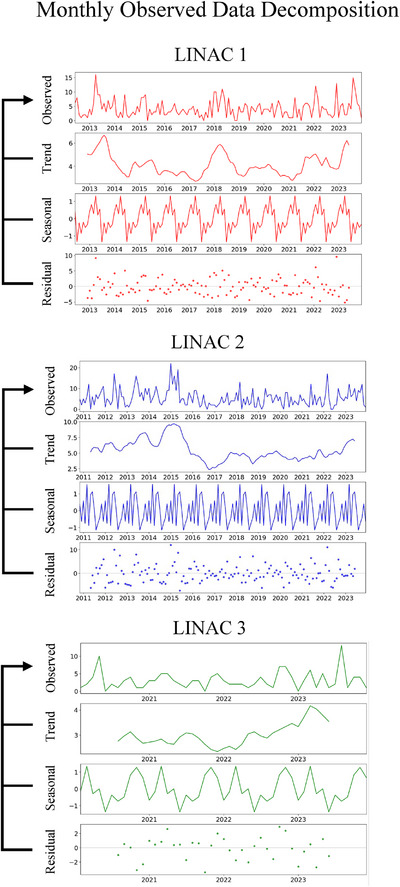
Decomposition of monthly downtime data into trend, seasonal, and residual components for each LINAC machine. Each column represents a different LINAC: LINAC 1 (red), LINAC 2 (blue), and LINAC 3 (green). The top row shows the original monthly observed data. The seasonal decomposition was performed using an additive model, where the sum of the trend, seasonal, and residual components reconstructs the original observed monthly data. The seasonal period was set to 12, reflecting the monthly frequency of the data. The unit on the *y*‐axis represents the number of reports.

Across all three LINAC machines, no consistent increasing or decreasing trend was observed over time.^18^ Seasonal strength calculated by Equation (2) showed weak seasonality for all machines, measuring 0.07 for LINAC 1, 0.05 for LINAC 2, and 0.21 for LINAC 3, significantly below the strong seasonality threshold of 0.7. Residuals for LINAC 1 and 2 showed occasional outliers, while LINAC 3 maintained relatively stable (Figure 4).^19^

(2) $$F_{s}=\max\left(0,1-\frac{Var\left(R_{t}\right)}{Var\left(S_{t}+R_{t}\right)}\right)$$

where $F_{s}=\mathrm{Strength}\;\mathrm{of}\;\mathrm{Sesonality}\left(\mathrm{between}\;0\;\mathrm{and}\;1\right)$, $Var(R_{t})=\mathrm{Variance}\;\mathrm{of}\;\mathrm{the}\;\mathrm{residual}\;\mathrm{component}$, and $Var(S_{t}+R_{t})=\mathrm{Variance}\;\mathrm{of}\;\mathrm{the}\;\mathrm{sum}\;\mathrm{of}\;\mathrm{seasonal}\;\mathrm{and}\;residual\;components$.

**FIGURE 5:**
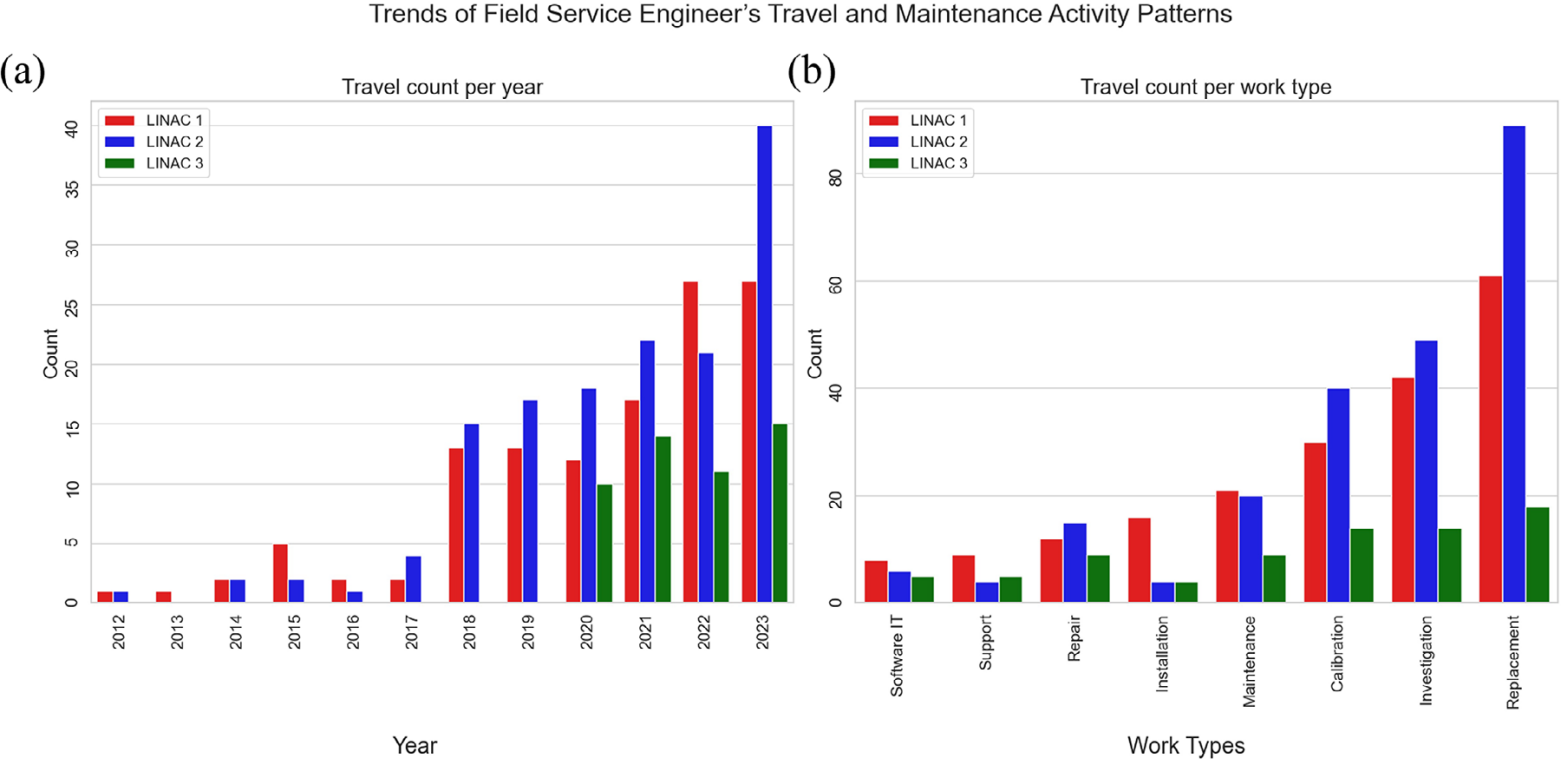
Trends and characteristics of maintenance activities requiring field service engineers’ (FSE) travel. (a) Shows annual count of service events involving travel, showing a rising trend across all LINACs. (b) Illustrates frequency of work type associated with travel‐based maintenance, indicating that replacement, investigation, and calibration are the most common services requiring FSEs.

**FIGURE 6:**
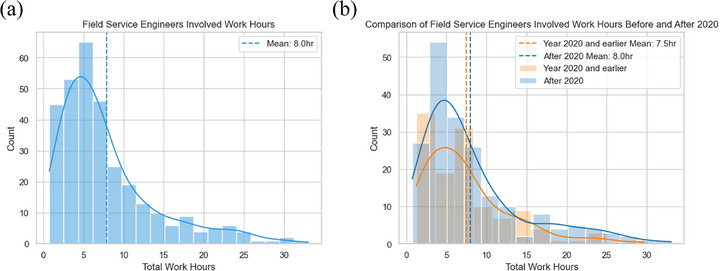
Distribution of work hours associated with maintenance activities involving field service engineers. (a) Overall distribution of work hours for all travel‐involved service events, showing a right‐skewed pattern with a mean of 8.0 h. (b) Comparison of work hour distributions pre and post‐ 2020 (used as a proxy for pre‐ and post‐COVID periods). Although the mean work hours increased from 7.5 h pre‐2020 to 8.0 h post‐2020, a Welch's two‐sample *t*‐test (*t* = 0.887, *p* = 0.376) indicated that this difference was not statistically significant.

**FIGURE 7:**
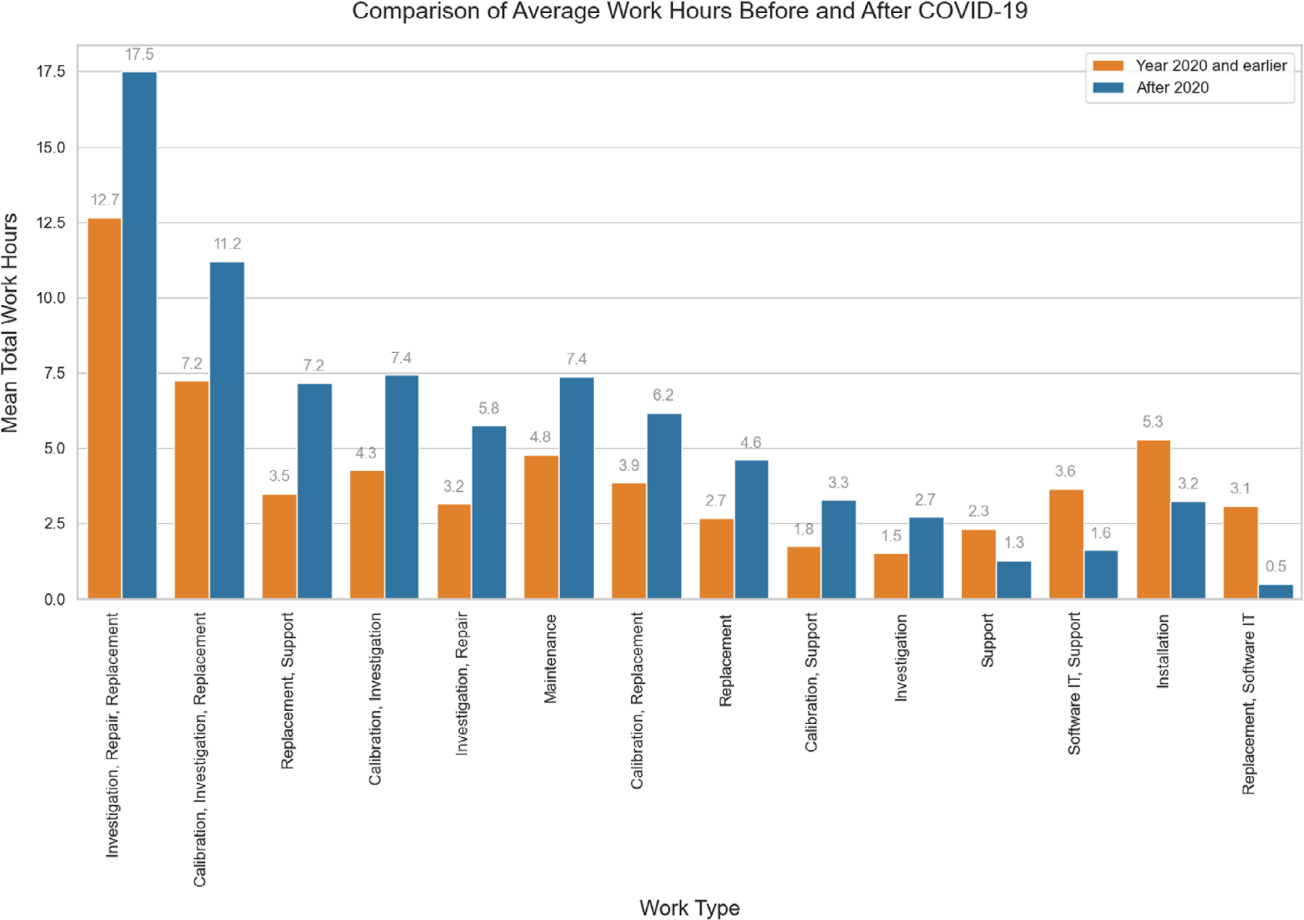
Comparison of mean total work hours by work type before and after the onset of the COVID‐19 pandemic. Orange bars represent average work hours per category for service reports dated 2020 and earlier, while blue bars represent those post‐2020.

In the analysis of work types requiring a vendor field service engineer (FSE), travel‐related maintenance events have increased steadily over the time, notably accelerating in recent years (Figure 5a). Replacement tasks most frequently required FSEs, followed by investigation and calibration (Figure 5b). Technician visits averaged around 8 h, occasionally exceeding 30 h (Figure 6a). A Welch's two‐sample *t*‐test was performed to compare total work hours before and after COVID‐19, yielding a *t*‐statistic of 0.887 and a *p*‐value of 0.376. Although the histogram suggests an increase in mean work hours from 7.5 to 8.0 h after 2020 (Figure 6b), this difference was not statistically significant. In the additional analysis of the impact of COVID‐19 on LINAC maintenance, the 14 most common work categories showed increased average work hours in 10 categories post‐2020, with the largest rises in investigation, repair, replacement involved work with 4.8 h, and calibration, investigation, replacement related work with 4.0 h (Figure 7). Decreased hours occurred in support and software IT‐related tasks.

## 4 DISCUSSION

AAPM TG‐100 describes a FMEA‐informed approach to the categorization of failures in radiation therapy and lists the common failure modes.^4^ Previous LINAC failure analyses often focused on specific components, such as the longitudinal analysis of ion chambers by Cetnar and DiCostanzo.^20^ More recent studies have expanded this scope to evaluate preventive and predictive maintenance strategies for LINACs in clinical settings. Rani et al. demonstrated that predictive maintenance frameworks based on historical service data can anticipate component‐level failures and improve treatment continuity.^21^ Dufek et al. conducted a multi‐institutional analysis of service records, highlighting the importance of structured maintenance scheduling to reduce unplanned downtime.^22^ Similarly, Gómez‐Casado et al. performed a statistical analysis of corrective maintenance events in radiotherapy LINACs, underscoring the operational impact of unscheduled interventions and the value of data‐driven maintenance planning.^23^

In contrast, this study provides a comprehensive analysis of overall LINAC performance. Downtime analysis confirmed the LINAC vendors' claims of 95% uptime. Overall, calculated downtime hours indicated stable and reliable LINAC performance over time. However, a significant peak occurred in 2021 for LINAC 2, potentially due to increased clinical utilization and backlog recovery following COVID‐19‐related service disruptions, placing heightened demand on equipment during that period. Our institution's dedicated in‐house LINAC engineering team likely contributes to reduced maintenance durations and expedited service case resolutions. Institutions lacking internal engineering resources may face higher maintenance workloads and prolonged downtimes, owing to their dependence on FSEs.

Analysis of failure and work type highlighted that collimation systems, control hardware, and power systems/distribution constitute a substantial proportion of service cases. Additionally, replacement and repair emerged as the most common work categories, emphasizing the importance of efficient inventory management in minimizing machine downtime by ensuring timely availability of necessary replacement parts. Optimizing the number of spare parts maintained in the institution's inventory can be a considerable point in reducing machine downtime hours and improving LINAC performance.

In the analysis of LINAC performance trends, an increase in failure frequency with prolonged machine usage was anticipated. However, the data did not demonstrate a clear upward trend in Figure 4. Instead, failure frequencies showed periodic fluctuations over certain years, possibly reflecting the stabilizing effects of regular software updates, patches or significant maintenance interventions that temporarily reduced malfunction rates following implementation.

It is important to note that direct comparison of downtime trends across the three machines is complicated by substantial differences in manufacturing vintage and design evolution. LINAC 1 was the 18th TrueBeam system installed clinically, while LINAC 2 was manufactured shortly thereafter, both are early “long stand” models that experienced manufacturing challenges common to first‐generation systems and operated on Version 1.0 software. In contrast, LINAC 3, manufactured approximately 3300 units later as a “short stand” model, benefited from years of accumulated design refinements, multiple software version updates, and significantly more complete technical documentation for FSEs. Notably, much of the technical documentation currently used by service representatives was developed based on troubleshooting experiences with early vintage machines, including our LINACs 1 and 2. These cumulative improvements contributed to enhanced reliability in later generation machines, which is reflected in the relatively lower downtime observed for LINAC 3 during its operational period. This generational difference should be considered when interpreting aging trends across the machines.

An increase in LINAC maintenance work hours was expected due to COVID‐19′s impact on supply chains. Consequently, records involving FSEs were analyzed separately, focusing on the “description” and “travel hours” columns in Table 1. This analysis identified a recent increase in FSE's involvement, particularly for replacement tasks, although the distribution of overall work hours did not significantly change. Further analysis comparing additional post‐2020 work type with pre‐2020 tasks showed that average work hours increased in most of the categories post‐2020, predominantly in replacement‐related activities. This trend may indicate heightened post‐pandemic service requirements, aging equipment, or the continuing impact of COVID‐19‐related supply chain disruptions. Conversely, reductions in work hours were noted in software IT and support categories, suggesting that vendors might have adopted more efficient or automated support systems during this period.

When interpreting the pre‐ and post‐2020 comparison, it is important to acknowledge that machine aging could potentially confound observed changes in maintenance patterns. However, COVID‐19‐specific impacts on downtime were minimal at our institution due to two protective factors. First, FSEs maintained uninterrupted site access throughout the pandemic. Second, and more importantly, our extensive spare parts inventory, systematically built based on historical failure pattern analysis, effectively mitigated supply chain disruptions. For example, because collimation systems consistently showed high failure frequency in our data, we maintain nearly all possible multi‐leaf collimator spare parts on‐site. When parts were back‐ordered during the pandemic, we utilized inventory stock and replenished later, avoiding treatment delays. While LINAC 2 shows increased downtime in recent years, as the oldest machine in our cohort manufactured early in the TrueBeam product cycle, these increases likely reflect aging effects rather than pandemic‐related disruptions. Other institutions without comparable spare parts inventory may have experienced more pronounced COVID‐19 impacts on maintenance operations.

The findings from this analysis have several practical implications for clinical operations, maintenance strategy, and budget management. First, comparison of the first four operational years across machines demonstrates clear improvements in both downtime frequency and maintenance incident rates between early‐generation (LINACs 1 and 2) and later‐generation (LINAC 3) systems. This information is valuable for institutions planning equipment replacement cycles and forecasting long‐term operational costs. Second, our maintenance approach, combining a full vendor service contract with a dedicated in‐house engineering team, enables rapid initial response and problem assessment, reducing overall downtime by beginning troubleshooting before FSE arrival. Third, our systematic approach to spare parts inventory management, guided by historical failure pattern analysis, proved instrumental in maintaining uptime. The collimation system analysis, which showed 22% of all maintenance cases, directly informed our decision to maintain comprehensive multi‐leaf collimator spare parts on‐site. This inventory strategy is reflected in our FSE travel data: the marked increase in FSE visits starting in 2018 coincides with our transition to a full‐service contract. Prior to 2018, we maintained an independent parts budget and purchased spares based on observed failure patterns, calling vendor support only when necessary. Under typical service contract models, vendors do not maintain extensive spare parts at client sites; however, since our inventory was established prior to contract transition, the vendor now replenishes parts as used. While maintaining an extended spare parts inventory represents greater upfront capital investment, this cost is minimal compared to the revenue impact of extended downtime on a clinical linear accelerator. These practical considerations including equipment generation planning, hybrid maintenance staffing models, and failure‐informed inventory management, represent actionable insights that other institutions can adapt to their specific operational contexts.

Several limitations should be acknowledged. First, beam‐on time and filament usage data were not systematically tracked in our institutional maintenance records across the study period. While some of this information exists in vendor‐generated reports, comprehensive extraction and standardization across a decade and three machines was not feasible for this analysis. Prospective collection of equipment utilization metrics (beam‐on time, filament usage, and patient treatment volume) alongside maintenance records would strengthen future analyses by enabling correlation between utilization patterns and failure frequency, particularly when comparing different operational periods or external factors such as the COVID‐19 pandemic.

This study was conducted at a single institution, which limits the generalizability of its findings and results may vary across different clinical environments. Nonetheless, the structured data‐processing methodology and the LLM‐based classification techniques developed here could be widely applied to other institutions with similar reporting practices. Additionally, the insights gained from this study would inform practical applications, such as clinical budget estimations based on malfunction frequencies and required FSEs visits. Future research should incorporate data from multiple institutions across diverse regions to improve the generalizability of results. Although this analysis did not reveal distinct trends or seasonality, larger datasets and additional variables such as patient treatment histories or detailed machine usage records could support the development of predictive malfunction models.

## 5 CONCLUSION

This study assessed the performance of LINACs over time by analyzing extensive historical maintenance records from three machines, identifying malfunction patterns, quantifying downtime durations, determinining the most vulnerable components, and evaluating the impact of the COVID‐19 pandemic on maintenance practices. Results indicated that all LINACs maintained downtime within the agreed threshold of 5%. In conclusion, insights from this research not only deepen the understanding of LINAC operational performance but also provide valuable implications for clinical budget management.

## AUTHOR CONTRIBUTIONS

Yeochan Youn contributed to study design, analysis and interpretation of data, and drafted the manuscript. Joseph B. Schulz contributed to data analysis. Ethan Stolen contributed to interpretation of data. Karl Ferry contributed to data collection and critical revision of the manuscript. Emil Muresan contributed to data collection. Siyoung Kim contributed to critical revision of the manuscript. James J. Sohn contributed to study design and drafted the manuscript.

## CONFLICT OF INTEREST STATEMENT

The authors declare no conflicts of interest.

## Figures and Tables

**FIGURE A1:**
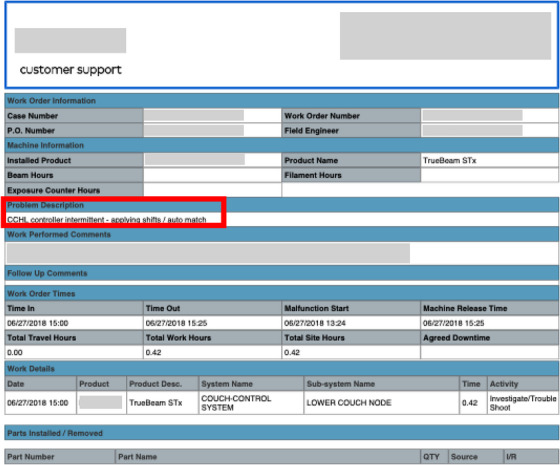
Sample LINAC service report and text extraction regions. Example of a LINAC service report in PDF format used in this study. Highlighted sections with red box indicate the textual regions extracted by the Python data pipeline: “Problem Description” (report subject). These sections were converted to text using PyMuPDF for subsequent analysis.

## APPENDIX A DATA PROCESSING WITH PYTHON PIPELINE.

### A.1

The LINAC service reports used in this study were stored in PDF format. The first step in processing these files for data analysis involved extracting the relevant information required for our study. To achieve this, we implemented a data processing pipeline in Python. The pipeline utilized PDFplumber (V0.11.5) and PyMuPDF (V1.26.5) to read and convert PDF files into text data. Python's built‐in regular expression (re) module was then used to extract specific text segments based on defined patterns.

For example, to extract the report subject beginning with the section title “Problem Description” in Appendix Figure A1, we applied the regular expression pattern:

r“Problem Description∖s+(.+?)(?:∖n|Work Performed Comments)”

for the subject. A detailed explanation of this regular expression pattern is provided in Appendix Table A1. A truncated sample output from the data pipeline after extracting all required fields is shown in Appendix Table A2.

**TABLE A1:** Regular expression patterns for extracting report sections. Summary of the regular expression (re) patterns used to identify and extract specific text sections from the LINAC service reports. The expressions capture text following each section header up to the defined stopping boundary.

ReGex component	Explanation	Example match
Problem Description	Matches the literal text “Problem Description”.	Problem Description
∖s+	Matches one or more whitespace characters (spaces, tabs, or newlines).	(newline after the heading)
(.+?)	Capturing group: matches any characters (non‐greedy), stops as soon as next part of pattern matches.	CCHL controller intermittent—applying shifts / auto match
(?:∖n	Non‐capturing group: matches either a newline ∖n or the literal phrase “Work Performed Comments”	Acts as a stopping boundary.

**TABLE A2:** Truncated sample output from the data processing pipeline. Example of structured text output generated by the Python data pipeline after extracting all required fields from a LINAC service report. The table shows key variables parsed from the original PDF, including machine identifier, problem description, service comments, event timestamps, and calculated work‐hour metrics.

Machine_id	Subject	Description	Malfunction_start	Machine_release	Total Work Hours	Total Site Hours
TrueBeam 2	CCHL controller intermittent—applying shifts / auto match	Reviewed event log, multiple 812602 faults …	06/27/2018 13:24	06/27/2018 15:25	0.42	0.42

## APPENDIX B LLM PROMPT DESIGN.

### B.1

Using the extracted text data (subject and description) from each LINAC service report, a large language model (LLM) was employed to classify long, unstructured text into predefined categories representing work type and failure type. To improve the accuracy and consistency of classification, multiple prompt engineering strategies were implemented within the Python script.

- Few‐shot prompting—Provides representative examples of input–output pairs to guide the model toward consistent classification behavior.

Example:

“”''
{“subject”: “GFIL, down”,
“description”: “”“Customer is getting intermittent GFIL …”“”
“failure_type”: “Beam Generation”},
{“subject”: “MLC is failing and secondary feedback”,
“description”: “”“Friday 6‐29‐2012: MLC down upon arrival, …”“”
“failure_type”: “Collimation System”},
…
“”''

- Chain‐of‐Thought (CoT) prompting—Encourages the model to reason step‐by‐step before providing a final label, improving accuracy of result and interpretability for ambiguous cases and.

Example:

“”''
### Failure Type Categories:
‐ “Beam Generation”: Components responsible for creating, …
Think carefully step by step and analyze the report content logically before classifying.
“”''

- Instruction prompting—Supplies clear, explicit instructions describing the classification task and expected output structure.

Example:

“”''
Each report can have multiple labels separated by comma. Your output must always be in dictionary format with key: `failure_type`.
“”''

- Role prompting—Assigns the model a specific role to align its responses with domain context.

Example:

“”''''
You are an expert medical engineering assistant who specializes in LINAC system troubleshooting.
“”''

- Output Formatting prompting—Enforces a standardized response schema (e.g., JSON) to facilitate downstream parsing and validation.

Example:

“”''
Return your result in JSON format only with key: `failure_type` and value: category (or comma‐separated categories) like:
{ “failure_type”: “Imaging System (KV/MV)”}
“”''

Full prompt and python implementation script used in the study are available online at https://github.com/YeochanY/LINAC‐Downtime‐Analysis/tree/main.
